# On the Elimination of Lead by Iodide of Potassium

**Published:** 1853-08

**Authors:** 


*On the Elimination of Lead by Iodide of Potassium.* By E. A. PARKES, M. D., Professor of Clinical Medicine in University College. (Supplement to a Memoir by M. MELSENS, on the use of Iodide of Potassium in Mercurial and Saturnine Poisoning.)

In the memoir of M. Melsens, so ably translated by Dr. Budd, in the last number of this journal, the statement, that the compounds formed by the union of mercury and its salts with certain of the tissues, can be destroyed, and the metal be dissolved by iodide of potassium, and be eliminated through the kidneys, is proved not only by clinical testimony, but by actual chemical evidence of the presence of mercury in the urine. The elimination of lead in the same way is rendered highly probable by the solubility of the saturnine salts and compounds in iodide of potassium, and by the undoubted prophylactic and curative powers of iodide of potassium in cases of impending or actual lead-poisoning. M. Melsens did not, however, chemically prove that lead could be made to pass off by the urine in the same way as is undoubtedly the case with mercury, and he left, therefore, a gap in the chain of evidence for future observers to fill up. A case of saturnine paralysis has lately occurred to the writer, in which iodide of potassium appeared to cause the elimination of lead in the urine—a fact which seems to complete the argument of M. Melsens.

A painter, aged 38, was admitted into University College Hospital, in February, 1853; he nad suffered for more than two years with paralysis of the extensors, and in a less degree, of the flexors, of both fore-arms; there was a well-marked blue line along the edge of the gums. He had been incapable of work for eighteen months, and had therefore not been exposed for a long time to any fresh source of poisoning. He has been treated for two months very carefully, but ineffectually, in the Middlesex Hospital, and, among other means, by "sulphur baths."

Professor Williamson was so good as to undertake the examination of the urine for lead, in the Birkbeck Laboratory of University College. He was furnished with four specimens: 1st. The urine of February 2d to 3d, no medicine having been given. 2d. The urine of February 3d to 4th, no medicine having been given. 3d. The urine of February 4th to 7th, 70 grains of iodide of potassium having been taken. 4th. The urine of February 7th to 10th, 90 additional grains of iodide of potassium having been taken.

Lead was not detected in the first two specimens of urine, but it was found in the urine passed after the employment of the iodide of potassium. I subjoin the report from the Birkbeck Laboratory:

"Four different portions of urine were received; two voided before the iodide was given, two voided afterward.

"Equal portions of the urine, Nos. 1 and 2, were evaporated to dryness; the black mass which remained was calcined, and the fused salt was boiled with excess of chlorine water. This treatment was adopted in order to get evidence of lead from the insoluble sulphate. The solution with chlorine was tested carefully for lead, but none could be detected.

"The portions of urine voided after the medicine were treated as follows:

"About a pint of the urine was evaporated, and the organic matter destroyed by aqua regia, and the remaining salt fused, and boiled for some time with carbonate of soda. After having collected the precipitate and undissolved portion, it was well washed, and then treated with dilute nitric acid. The filtered solution was tested for lead with sulphuretted hydrogen, and it yielded a black precipitate of sulphide of lead. From the sulphide of lead from one of the urines, a distinct, though a very minute, metallic globule of lead was obtained.

"The quantities of lead present in the urine Nos. 3 and 4, seemed to be about equal, but too small for quantitative estimation.

"BIRKBECK LABORATORY, March 3d, 1853."

It may possibly still be questioned whether lead might not have been detected before the use of the iodide, had the second and more delicate process been employed. This is, however, unlikely, for not only was the first process a very good one, but it can hardly be conceived that lead could have been passing off daily with the urine, before the employment of the iodide, without some improvement having taken place in the symptoms. So far from the symptoms having improved, they had been quite stationary for a long time, as usually observed in this obstinate form of paralysis. The compounds formed by lead with the tissues are well known to be extremely stable; and judging merely from the duration of the disease, the normal disintegration of the tissues appears in most cases quite insufficient to cause the elimination of the metal.

The iodide of potassium was administered in ten-grain doses, and on an empty stomach, in order to prevent decomposition by acids—a change which appears to destroy half its power. It was intended to combine galvanism with it, but unfortunately the patient having behaved improperly was obliged to be dismissed from the hospital. At the date of discharge no improvement in the symptoms was apparent.

The only other points determined in respect of the urine were the influence of the iodide on the water and sulphuric acid.

*Effects of Iodide of Potassium on the Water and Sulphuric Acid.*

| Date. | Medicine. | Urine, quantity in 24 hours. | Reaction. | Sulphuric acid in urine in 24 hrs. | Remarks. |
|---|---|---|---|---|---|
| Feb. 2, 3 | None . . . | ℥46 | Acid. | 23·46 | |
| " 3, 4 | " . . | ℥44 | " | 31·90 | |
| " 4, 5 | Ha'st.purgans, pot. iod. grs. x. | ℥18 | " | 24·426 | Several very loose stools, no urine with them; lithates. |
| " 5, 6 | Pot. iod., ʒss. | ℥32 | " | 15·520 | |
| " 6, 7 | " ʒss. | ℥54 | " | 26·784 | |
| " 7, 8 | " ʒss. | ℥40 | " | 27·08 | Commenced to be iodized. |
| " 8, 9 | " ʒss. | ℥48 | " | 33·536 | Strongly iodized. |
| " 9, 10 | " ʒss. | . . | . . | . . | Some urine lost by mistake, 39 oz. measured. |
| " 10, 11 | None . . . | ℥33½ | " | 26·197 | |
| " 11, 12 | " . . | ℥40 | " | 20·760 | Effects of iodide pass'g off. |
| " 12, 13 | Haust. purgans | . . | . . | . . | Some stools on the 12th, urine with them. |
| " 13, 14 | None . . . | ℥54 | . . | 30·076 | Not iodized. |
| " 14, 15 | Pot. iod., ℈j. | ℥55½ | " | 30·305 | Galvanism. |
| " 15, 16 | " ℈ij. | ℥54 | " | 28·134 | Galvanism. |

The iodide, in doses of 30 grains in 24 hours, did not seem to have much effect on the water, and very little on the sulphuric acid. It is probable, indeed, that in most cases, this remedy has not the disintegrating and destructive effect of the alkaline salts of potash, although it possibly heightens this effect when combined with them.

The singular decrease in the quantity of water on the 4th, 5th, and of the sulphuric acid on the 5th, 6th, is an interesting point, as it forms the fourth instance in which a striking decrease of sulphuric acid in the urine has been noticed after catharsis. The coincidence is worthy of inquiry, although it may turn out, on a more extended examination, to have been merely accidental.—*British and Foreign Medico-Chir. Review.*

